# Age at Menopause and Mortality in Japan: the Jichi Medical School Cohort Study

**DOI:** 10.2188/jea.16.161

**Published:** 2006-07-13

**Authors:** Yoko Amagai, Shizukiyo Ishikawa, Tadao Gotoh, Kazunori Kayaba, Yosikazu Nakamura, Eiji Kajii

**Affiliations:** 1 Division of Community and Family Medicine, Center for Community Medicine, Jichi Medical University.; 2 Wara National Health Insurance Hospital.; 3 School of Health and Social Services, Saitama Prefectual University.; 4 Department of Public Health, Jichi Medical University.

**Keywords:** Cohort studies, Mortality, Menopause, Menopause, Premature, Japan

## Abstract

**BACKGROUND:**

Although several studies have reported increased mortality risk with early menopause, there were no studies examining the relationship between age at menopause and mortality in Japan. The goal of this analysis is to investigate the relationship between age at menopause and all-cause mortality among the Japanese.

**METHODS:**

Study subjects were 4,683 postmenopausal females in the Jichi Medical School Cohort Study, a population-based prospective study. Baseline data were obtained by questionnaire and health checkups between April 1992 and July 1995 in 12 rural areas in Japan. Main outcome measures were all-cause mortality derived from death certificates up to December 31, 2002. Cox’s proportional hazard models were used to analyze the association of age at menopause with mortality.

**RESULTS:**

A total of 215 deaths were observed during the average of 9.2 year follow-up period. After adjusting for age, systolic blood pressure, serum total cholesterol level, serum high density lipoprotein cholesterol level, history of diabetes mellitus, body mass index, smoking habits, alcohol drinking habits, marital status, study area, and types of menopause, the hazard ratios (95% confidence intervals) of all-cause mortality were 2.10 (1.07-4.11), 0.68 (0.36-1.26), 0.94 (0.68-1.30), and 1.17 (0.63-2.20) for females with a menopause at ages younger than 40 years, 40-44, 50-54, and 55 or older, respectively, relative to those with menopause at age 45-49 years.

**CONCLUSIONS:**

Our data suggest that menopause aged younger than 40 years increases the risk of death from all causes among the Japanese.

Menopause occurs by loss of ovarian function and subsequent deficiency of endogenous estrogens. Relationship between age at menopause and mortality is mainly explained by deficiency of endogenous estrogens. Many epidemiologic studies indicated that an early natural menopause increased the risk of ischemic heart disease.^1^^-^^4^ Furthermore, a vast amount of observational studies suggested that hormone replacement therapy (HRT) protected postmenopausal women from cardiovascular disease.^5^ Many basic studies also suggested that estrogen had protective effects on the cardiovascular system.^6^^-^^9^ However, randomized controlled trials^10^^-^^12^ did not show the effect of HRT on cardiovascular disease outcome. On the other hand, some epidemiologic studies reported that later menopause increased the risk of breast cancer^13^^,^^14^ and endometrial and ovarian cancer,^15^^,^^16^ and many studies reported that HRT also increased the risk of breast cancer.^12^^,^^17^

There were some studies examining the relationship between age at menopause and all-cause mortality. Most previous reports have found inverse relationship between age at menopause and all-cause mortality.^18^^-^^21^ In other words, mortality of those with menopause at young age was higher than the mortality of those with menopause at old age. To our knowledge, all previous studies were conducted in Western countries, and there was no study in Japan.

The purpose of this study was to examine the relationship between age at menopause and all-cause mortality in the Japanese population.

## METHODS

We used the data set of the Jichi Medical School Cohort Study, which is a population-based prospective study investigating risk factors for cardiovascular diseases starting in 1992.^22^ The baseline data were obtained between April 1992 and July 1995 in 12 rural areas in Japan. Mass screening examinations for cardiovascular diseases have been conducted since 1983 in accordance with the Health and Medical Service Law for the Aged of 1982, and we used this system to collect the data. The subjects for the mass screening examinations were residents aged 40-69 years in 8 areas, and were 30 years and older in one area. Subjects for other age groups were included in 3 areas. In each community, a local government office sent personal invitations to all the subjects by mail.

Health checkup was carried out in each community. Body mass index (BMI) was calculated as weight (kg) / height (m)^2^. Systolic blood pressure (SBP) and diastolic blood pressure (DBP) were measured with a fully automated sphygmomanometer, BP203RV-II (Nippon Colin, Komaki, Japan). Serum total cholesterol and high density lipoprotein (HDL) cholesterol were also measured with standard methods, as reported previously.^22^

Information about medical history and sociodemographic characteristics was obtained by trained interviewers using a standardized questionnaire. Smoking status was judged as current smoking, ex-smoking, or never smoking, and alcohol drinking status was judged as current drinking, ex-drinking, or never drinking. Females were asked about the age at menopause and the type of menopause (natural or surgical). We did not show the definition of the word of menopause, and we did not ask about HRT use.

A total of 12,490 subjects (4,911 males and 7,579 females) participated in the mass screening and the response rate for the residents who underwent a basic medical checkup was about 99%. The proportion of people who actually underwent a medical checkup for the total population was 65.4%. Written informed consent for the study was obtained individually for the responders of the mass screening, and 95 responders (38 males and 57 females) rejected to join the study. Therefore, 12,395 subjects (4,873 males and 7522 females) who were 19-93 years of age agreed to participate in the study. For our purpose, we identified 4,848 females with data of age at menopause, and we excluded those who reported a previous history of myocardial infarction (24 females), stroke (47 females), and malignant neoplasms (87 females). A total of 4,683 females aged between 36 and 89 years remained.

Causes of death were identified using death certificates, which were collected at the respective local public health centers with permission from the Ministry of Internal Affairs and Communications. The people who moved out of the communities during the observation period were followed up until the date of the emigration. Data on emigration of the study subjects were obtained every year from their municipal governments.

Age at menopause was categorized into <40 years, 40-44 years, 45-49 years, 50-54 years, and 55+ years. The reference group was females who were 45-49 years at menopause.

Statistical analysis was performed using the SPSS^®^ software version 11.0. Descriptive parameters are shown as mean, standard deviation or proportion (%). We compared characteristics between the groups by one-way analyses of variance or the chi-square tests. Cox’s proportional hazard models were used to calculate hazard ratios of mortality for age at menopause after adjusting for age, SBP, serum total cholesterol level, HDL cholesterol level, history of diabetes mellitus, BMI, smoking habits, alcohol drinking habits, marital status, study area, and type of menopause, which were considered to be potential confounding factors. Among the potential confounding factors that we obtained as the baseline data about blood pressure, we used SBP only in the multivariate analyses to avoid the co-linearity.

This study was approved by the Institutional Review Board of Jichi Medical School for ethical issues.

## RESULTS

The total observed person-years were 43,152, and the mean follow-up period±standard deviation (SD) was 9.2±1.6 years. The mean age±SD was 61.0±6.7 at baseline data collection. There were 215 deaths (4.6%) during the study period. Of these, 93 died of malignant neoplasms, 29 of heart diseases, 29 of stroke, and 64 of other diseases. The mean age±SD at menopause were 48.3± 4.8; 80% of females had their menopause between 45 and 54 years of age. Baseline characteristics in relation to each category of age at menopause are shown in Table 1. The mean age at baseline data collection, SBP, DBP, and serum total cholesterol level tended to be higher among those with old age at menopause. Females with early menopause were more likely to smoke and have surgical menopause.

**Table 1.** Baseline characteristics according to age at menopause.

	Age at menopause (year)					P-value

	<40	40-44	45-49	50-54	55+	
N (%)	227 (5)	467 (10)	1629 (35)	2124 (45)	236 (5)	
Age at baseline data collection (year)	56.5 (9.6)	60.0 (7.7)	60.9 (6.6)	61.6 (6.0)	62.9 (4.7)	< 0.001*
Systolic blood pressure (mmHg)	128.7 (21.3)	130.5 (21.5)	130.6 (21.0)	133.1 (20.6)	134.8 (19.0)	< 0.001*
Diastolic blood pressure (mmHg)	77.5 (12.0)	77.5 (12.2)	77.2 (11.9)	78.6 (11.7)	79.3 (10.8)	0.003*
Total cholesterol (mg/dL)	199.7 (34.8)	201.9 (35.5)	202.7 (33.0)	205.0 (32.6)	209.4 (35.0)	0.003*
High density lipoprotein cholesterol (mg/dL)	50.6 (11.5)	52.1 (12.5)	52.3 (12.2)	52.4 (12.8)	51.6 (12.8)	0.275*
Body mass index (kg/m^2^)	23.6 (3.7)	23.3 (3.2)	23.2 (3.2)	23.3 (3.1)	24.0 (3.4)	0.014*
History of hypertension(%)	18.5	20.3	20.6	21.1	23.3	0.764^†^
History of diabetes mellitus(%)	5.3	3.9	3.6	3.8	5.1	0.618^†^
Current smokers (%)	6.4	4.8	4.5	3.1	2.6	< 0.001^†^
Current alcohol drinkers (%)	21.4	21.8	19.2	20.6	21.4	0.702^†^
Having a spouse(%)	93.8	89.4	90.7	91.2	91.5	0.403^†^
Type of menopause(%)						0.031^†^
Natural	18.9	57.4	82.6	90.5	91.9	
Surgical	77.1	38.8	12.3	3.1	2.1	
No answer	4.0	3.9	5.1	6.4	5.9	

Mean values (standard deviation) for quantitative data and proportion in percentage for qualitative data are shown.

* : Analysis of variance (ANOVA)

† : Chi-square test (degree of freedom = 4)

Table 2 presents crude mortality rates and the two kinds of hazard ratios for all-cause mortality by age category at menopause. The crude mortality rate was the highest for the youngest group, and was the lowest for those with menopause during 40 to 44 years of age. Compared to females who were 45-49 years of age at menopause, the age-adjusted hazard ratio was not significantly but higher for females with a menopause at age younger than 40 years. There were 6 deaths among females who had natural menopause before 40 years. These causes of deaths were acute myocardial infarction, cerebral infarction, malignant lymphoma, gingival cancer, bile duct cancer, and pneumonia. After further adjustment for SBP, serum total cholesterol level, serum HDL cholesterol level, history of diabetes mellitus, BMI, smoking habits, alcohol drinking habit, marital status, study area, and type of menopause, the results remained essentially unaltered, but the highest hazard ratio for the youngest age group became significant. The crude mortality rate and the two kinds of hazard ratios were essentially same as above among who had a natural menopause. The multivariate-adjusted hazard ratios of those with surgical menopause at younger age were higher than those with surgical menopause at older age, in spite of the lack of significance. We analyzed in the same way in those who were age of 55 years and older at baseline, then the results were not so different.

**Table 2.** Association between age at menopause and all-cause mortality according to type of menopause: crude mortality rate and adjusted hazard ratios.

Type of menopause	Age at menopause (year)	Number of death (death/total (%))	Crude mortality rate*	All subjects		Aged 55+ years^†^	
	
				HR-age	HR-all	HR-age	HR-all
				(95% CI)	(95% CI)	(95% CI)	(95% CI)
All	< 40	14 / 227 (6.2)	6.8	1.72 (0.97-3.03)	2.10 (1.07-4.11)	1.39 (0.72-2.69)	1.64 (0.76-3.54)
	40- 44	15 / 467 (3.2)	3.5	0.67 (0.39-1.17)	0.68 (0.36-1.26)	0.72 (0.41-1.26)	0.71 (0.38-1.33)
	45- 49	78 / 1629 (4.8)	5.2	1.00 (reference)	1.00 (reference)	1.00 (reference)	1.00 (reference)
	50- 54	96 / 2124 (4.5)	4.9	0.87 (0.65-1.18)	0.94 (0.68-1.30)	0.85 (0.62-1.16)	0.90 (0.64-1.27)
	55+	12 / 236 (5.1)	5.5	0.98 (0.54-1.80)	1.17 (0.63-2.20)	1.04 (0.57-1.93)	1.26 (0.67-2.39)

Natural	< 40	6 / 43 (14.0)	16.3	2.75 (1.19-6.36)	2.77 (1.16-6.58)	2.34 (0.94-5.84)	2.20 (0.86-5.65)
	40- 44	10 / 268 (3.7)	4.0	0.67 (0.34-1.30)	0.53 (0.24-1.17)	0.67 (0.34-1.31)	0.53 (0.24-1.18)
	45- 49	63 / 1346 (4.7)	5.1	1.00 (reference)	1.00 (reference)	1.00 (reference)	1.00 (reference)
	50- 54	89 / 1923 (4.6)	5.0	0.91 (0.66-1.25)	0.94 (0.67-1.33)	0.85 (0.61-1.18)	0.88 (0.62-1.26)
	55+	12 / 217 (5.5)	6.0	1.16 (0.63-2.16)	1.39 (0.73-2.63)	1.19 (0.64-2.20)	1.41 (0.74-2.69)

Surgical	< 40	7 / 175 (4.0)	4.4	0.92 (0.35-2.41)	1.58 (0.46-5.39)	0.95 (0.30-3.01)	1.30 (0.29-5.80)
	40- 44	4 / 181 (2.2)	2.4	0.48 (0.15-1.53)	1.04 (0.27-4.06)	0.68 (0.20-2.34)	1.31 (0.29-5.94)
	45- 49	10 / 200 (5.0)	5.5	1.00 (reference)	1.00 (reference)	1.00 (reference)	1.00 (reference)
	50- 54	1 / 66 (1.5)	1.6	0.27 (0.04-2.14)	0.46 (0.05-4.26)	0.37 (0.04-2.99)	0.45 (0.04-4.89)
	55+	0 / 5 (0.0)	-	-	-	-	-

HR-age: hazard ratios adjusted for age.

HR-all: hazard ratios adjusted for age, systolic blood pressure, total cholesterol, high density lipoprotein cholesterol, history of diabetes mellitus, body mass index, smoking habits, alcohol drinking habits, marital status, study area, and type of menopause.

- : no death in the category.

* : per 1000 person-year

† : Subjects excluded whose baseline ages were before 55 years.

To eliminate the influence of potentially preexisting subclinical diseases, we repeated analyses excluding those who died within 2 years after the baseline examination, and the results were essentially unchanged (data not shown).

## DISCUSSION

We investigated the association between reported age at menopause and all-cause mortality using data from the Jichi Medical School Cohort Study, a population-based cardiovascular cohort study. The present study did not show an inverse relationship between age at menopause and all-cause mortality, but comparing females with early menopause (<40 years) with those who experience menopause when they were 45-49 years old, the former group has significantly higher all-cause mortality.

There were several epidemiologic studies examining the relationship between age at menopause and all-cause mortality. According to Ossewaarde et al^18^ studying in a breast cancer screening cohort comprising 12,134 postmenopausal Dutch females followed for average of 17 years, a later menopause was associated with a decreased all-cause mortality risk, showing a linear trend. Jacobsen et al^19^ reported there was a weak inverse relationship between age at natural menopause and all-cause mortality in a cohort of 19,731 Norwegian postmenopausal females followed for average of 37 years. Cooper et al^20^ also reported an inverse relationship between age at natural menopause and all-cause mortality in the National Health and Examination Survey Epidemiologic Follow-up Study followed up 3,191 postmenopausal females during a 4-year period. Jacobsen et al^21^ studied the relationship between age at natural menopause and all-cause mortality in a cohort of 6,182 California Seventh-Day Adventist females who reported a natural menopause followed for average of 13 years. The result was same as above.

The average age for menopause is approximately 49 years in previous studies in Western countries.^18^^-^^21^ Premature ovarian failure (POF) or premature menopause refers to development of amenorrhoea due to cessation of ovarian function before the age of 40 years. POF occurs in 1% of females.^23^^-^^25^ In our study, limiting natural menopause, 1.1% of females reported to menopause <40 years, same as former studies, and they had significantly higher risk of death than those reported to menopause 45-49 years. A wide spectrum of pathogenic mechanisms may lead to the development of POF including chromosomal,^26^ genetic,^27^^,^^28^ autoimmune,^29^^,^^30^ metabolic,^31^ infectious,^32^^-^^34^ and iatrogenic causes.^35^^,^^36^ It may result in the higher mortality on early menopausal females.

Observational studies have shown a reduced risk of cardiovascular disease,^1^^-^^4^ fewer calcifications in the aorta,^37^ and less extensive atherosclerosis^38^ in females with later menopause. In contrast, later menopause has been related to increased risk of breast cancer,^13^^,^^14^ endometrial and ovarian cancer.^15^^,^^16^ We analyzed cause specific mortality, but no significant relationship was seen in any category. That was because the number of deaths in our analysis was too small to draw firm conclusions about specific causes of death. We cannot explain why our result did not show an inverse relationship between age at menopause and all-cause mortality, especially why females who had menopause 40-44 years had lower mortality, though it was not significant, than those who had menopause 45-49. All the previous studies were conducted in Western countries in which major cause of death is cardiovascular disease. On the other hand, major cause of death was malignant neoplasms in Japan. It may influence our result. To clarify these questions, cause specific mortality will be useful. We are going to analyze cause specific mortality after extending the follow-up period.

In our study, the multivariate-adjusted hazard ratios of those with surgical menopause at younger age were higher (though not significantly) than those with surgical menopause at older age. Although we did not ask whether both ovaries were still present or not, we excluded those who had a history of malignant neoplasms from the subject of our study. Therefore, many females who had surgical menopause may have both ovaries. We did not know why the younger group had higher mortality. The subjects of many previous studies were limited only natural menopause, the relationship between surgical menopause and mortality in not well known.^19^^-^^21^ Ossewaarde et al^18^ reported type of menopause (natural menopause or not) did not affect the associations between age at menopause and cause specific mortality. Some other factors except ovarian function may influence the association between age at menopause and mortality.

Our current data suggested that those with menopause at young age have a high risk of mortality. Although the cause-specific analyses are required, health management, such as an intensive health check-up system, may be useful for such females.

The strong points of our research are as follows. First, to our knowledge, our report was the first in Japan. Second, we adjusted for possible confounding factors such as age, SBP, serum total cholesterol level, serum HDL cholesterol level, history of diabetes mellitus, BMI, smoking habits, alcohol drinking habits, marital status, and study area, and conducted this research with an adequately accurate method of measurement. Accordingly, we aimed at thorough unification of the methodology, such as blood pressure measurement and blood testing. In order to eliminate variation arising from individual techniques, the same automatic sphygmomanometer was used in all the areas. Furthermore, the blood samples were analyzed at a single trusted laboratory using the same method of measurement.

There are three limitations in our study. First, this study makes biased reporting of age at menopause unlikely. However, as demonstrated in previous reports, self-reported age at menopause and type of menopause are reasonably valid and reproducible.^39^^,^^40^ Second, we did not ask about HRT use. However, there are few females who take HRT in Japan, and we think the influence of HRT was small.

The final limitation is that those who were before menopause at the baseline data collection were excluded from our data analyses. This restriction might lead a selection bias, which is that we observed only those whose age at menopause was relatively younger. However, the results were not so different in the analyses with subjects aged 55 years and older; most of them were considered menopausal condition.

In conclusion, our data indicated that menopause aged younger than 40 years increases the risk of death from all causes among Japanese.
